# Impact of Culture and Religion on End-of-Life Decisions Among Advanced Cancer Patients in Developing Countries

**DOI:** 10.1093/geroni/igaa057.787

**Published:** 2020-12-16

**Authors:** Justina Yevu Johnson, Lori Popejoy

**Affiliations:** 1 University of Missouri, Columbia, Columbia, Missouri, United States; 2 University of Missouri, Columbia MO, Columbia, Missouri, United States

## Abstract

Palliative care and end of life decisions are important components of quality care at the end-of-life. Individual’s perception of cancer diagnosis is affected by their customs and traditions, religious orientations and stigma. Culture and religion as a social determinant of health affects people’s interpretation of health and illness and is a major factor in deciding the type of care at end of life and death. The purpose of the review was to identify factors related to culture and/or religion that impact decision making at end of life among advanced cancer patients their primary family caregivers and healthcare providers. An extensive literature search was conducted in Psych Info, PubMed, Philosophy Index, Atlas Religion, and Academic Search Premier databases for primary studies on the topic. Primary studies conducted only in developing countries and among healthcare providers, advanced cancer patients and their primary family caregivers were included. Five studies met the inclusion criteria: two primary studies, one methodological paper, and two on perspectives. The studies reported economic status of the patient, family, culture, and religious beliefs as factors that affected decision making at the end of life. Improving cancer care in developing countries requires the accommodation of the culture, traditions, and religious beliefs of both healthcare providers, patients and family. Culturally appropriate care model is therefore needed to enhance palliative and end of life care in developing countries. Leininger’s Cultural Care Theory seem an appropriate path to take.

